# Studying the mechanisms of neurodegeneration: *C. elegans* advantages and opportunities

**DOI:** 10.3389/fncel.2025.1559151

**Published:** 2025-03-26

**Authors:** Angie K. Torres, Rodrigo G. Mira, Cristina Pinto, Nibaldo C. Inestrosa

**Affiliations:** ^1^Centro de Excelencia en Biomedicina de Magallanes (CEBIMA), Escuela de Medicina, Universidad de Magallanes, Punta Arenas, Chile; ^2^Departamento de Biología Celular y Molecular, Facultad de Ciencias Biológicas, Pontificia Universidad Católica de Chile, Santiago, Chile

**Keywords:** *C. elegans*, neurodegeneration, models, genetic modulation, pharmacological treatment, advantages and disadvantages

## Abstract

*Caenorhabditis elegans* has been widely used as a model organism in neurodevelopment for several decades due to its simplicity, rapid growth, short life cycle, transparency, and rather simple genetics. It has been useful in modeling neurodegenerative diseases by the heterologous expression of the major proteins that form neurodegenerative-linked aggregates such as amyloid-*β* peptide, tau protein, and *α*-synuclein, among others. Furthermore, chemical treatments as well as the existence of several interference RNA libraries, transgenic worm lines, and the possibility of generating new transgenic strains create a magnificent range of possible tools to study the signaling pathways that could confer protection against protein aggregates or, on the contrary, are playing a detrimental role. In this review, we summarize the different *C. elegans* models of neurodegenerative diseases with a focus on Alzheimer’s and Parkinson’s diseases and how genetic tools could be used to dissect the signaling pathways involved in their pathogenesis mentioning several examples. Finally, we discuss the use of pharmacological agents in *C. elegans* models that could help to study these disease-associated signaling pathways and the powerful combinations of experimental designs with genetic tools. This review highlights the advantages of *C. elegans* as a valuable intermediary between *in vitro* and mammalian *in vivo* models in the development of potential new therapies.

## 1 Introduction

*C. elegans* is a free-living millimetric-sized organism that has become an outstanding model of study in biology. *C. elegans* belongs to the phylum Nematoda, which are commonly known as roundworms (Strange, 2006). This nematode was isolated for the first time in 1900 by Emile Maupas and was published for the first time as a model organism in 1974 by the team of Brenner (1974) and Nigon and Felix (2017). Since then, the anatomy of the *C. elegans* has been widely studied, and now it is well described. The body wall of their cylindrical body consists of a collagen cuticle with hypodermis, muscles, and nerves beneath. The mature adult hermaphrodite nematodes have 959 somatic cells, while males have 1,031 (Strange, 2006). These somatic cells had a high degree of differentiation, allowing many mammals’ physiological processes to have their homolog in *C. elegans* (Strange, 2006). The nervous system of these nematodes is also well described and consists of 302 neurons and 56 glial cells in hermaphrodites and 381 neurons and 92 glial cells in males, which correspond to almost a third of the total number of cells in the organism. Most neuronal somas are located in the head of the worms clustered in the ganglia. Interestingly, despite the compact nervous system, compared with other organisms, there is a great variety of neuron types (118 for hermaphrodites) that allows this tiny organism to perform complex behaviors mostly related to sensory processes (White et al., 1986). Indeed, genome sequence analysis shows that nearly 80% of human genes have a homolog in *C. elegans* (Consortium, 1998), which made this animal a great model for studying different human diseases. In fact, it has been used for studies in different areas such as molecular biology, development, obesity, neurobiology, aging, and neurodegenerative diseases (Corsi et al., 2015; Shen et al., 2018; Sorrentino et al., 2017).

The maintenance of these nematodes is quite easy and cheap since they are usually cultured in Nematode Growth Medium (NGM) agar plates, or they also can be maintained in a liquid medium. In both cases they are fed with *Escherichia coli* (*E. coli*), where the most common *E. coli* used is the strain OP50 (Stiernagle, 2006). Moreover, one great characteristic is their short life cycle, which involves an embryonic stage, four larval stages known as L1-L4, and adulthood (Shen et al., 2018). The maintenance of these nematodes in the laboratory is usually at 20°C, where the transition from eggs to L4 stage occurs in 48 h, while from L4 to adulthood is in 24 h (Ewald et al., 2018). Hence, the reproductive cycle of *C. elegans* is approximately 3 days, while their mean lifespan is about 20 days (Ewald et al., 2018; Zhang et al., 2020). However, they could also live at 15°C where the growth is slower, or at 25°C where the growth is faster (Gomez-Orte et al., 2018; Klass, 1977). These changes in the growth conditions with temperature led to the discovery of temperature-sensitive genes due to the presence of temperature-sensitive promoters, which have been an outstanding tool in the development of inducible gene-expression strains (Bacaj and Shaham, 2007; Link et al., 2003). Interestingly, if the maintenance conditions are unfavorable, for example, high population density or low food availability, *C. elegans* can enter a developmentally arrested stage known as the Dauer larval stage (Thomas, 1993; Zhang et al., 2020). Dauer stage is a non-feeding alternative L3 stage induced by the dauer pheromone, which is produced through the entire life cycle of *C. elegans* inhibiting proper development (Bargmann and Horvitz, 1991; Butcher et al., 2008; Golden and Riddle, 1982; Thomas, 1993). Morphological, behavioral, and metabolic adaptations occur during dauer stage which allows *C. elegans* to survive in hostile environments even for months (Thomas, 1993), facilitating the culture conditions of these worms in the laboratory. When the environmental conditions improve, they could return to normal development to the L4 larval stage with no change in their lifespan (Fielenbach and Antebi, 2008; Thomas, 1993). This characteristic of a short life cycle gives them advantages compared to traditional rodent models like mice. For example, for studying aging and neurodegenerative diseases, wild-type (WT) C57BL6/J mice must reach at least 18 months to be considered aged animals (Ackert-Bicknell et al., 2015; Torres et al., 2021a), compared to the 21 days life cycle of these worms.

Another beneficial characteristic of *C. elegans* is the size of their progeny. These nematodes can be hermaphrodites that self-fertilize themselves or males, although the majority are hermaphrodites since male appearance occurs at a frequency of less than 0.2% (Corsi et al., 2015). The difference between both sexes is that hermaphrodites have two X chromosomes (XX) and males only have one (XO) which occurs due to a rare meiotic non-disjunction of the X chromosome (Girard et al., 2007). For self-fertilizing, hermaphrodites during the L3 stage generate the spermatogenesis process in the gonad, producing sperm cells that are stored when reaching L4. Then, the gonad starts with oogenesis, producing oocytes that will be fertilized by the stored sperm cells which could produce a progeny of approximately 300 worms (Hester et al., 2017). When hermaphrodites are fertilized by male worms, the number of offspring increases to around 1,000 worms (Strange, 2006). The fast reproductive cycle with the high number of offspring makes the generation of new strains by crossbreeding of different *C. elegans* strains, an easier and faster process in comparison to mice.

Furthermore, these nematodes are easy to manipulate genetically (Katz, 2022; Sugi, 2017). Indeed, if the hermaphrodites are mutated by any genetic approach, this mutation will be present in the next generations due to self-fertilization without the need for mating (Grishok, 2005). The genetic manipulations in *C. elegans* and the fact that their whole genome is sequenced have resulted in a wide library of transgenic strains commercially available in the Caenorhabditis Genetics Center (CGC), which includes several neurodegenerative disease models such as amyotrophic lateral sclerosis (ALS), Alzheimer’s disease (AD), Parkinson’s disease (PD), and Huntington’s disease (HD) (Caldero-Escudero et al., 2024), which will be described in this review. Also, we will highlight the genetic and pharmacological strategies that are used in these strains that are useful to determine the role of proteins involved in different signaling pathways and cellular responses, as well as how they change in response to different stimuli in the context of neurodegenerative diseases.

## 2 *C. elegans* models of neurodegenerative diseases

Neurodegenerative diseases such as AD, PD, ALS, and HD are aging-related diseases, characterized by neuronal death of selectively vulnerable populations of neurons, and the accumulation of protein aggregates (Choonara et al., 2009; Dugger and Dickson, 2017). Furthermore, there are no long-term effective pharmacological treatments for these diseases, making it worth seeking new mechanisms and/or new valuable products with therapeutical potential (Lamptey et al., 2022). To understand the consequences of human genes related to neurodegenerative diseases, the nematode *C. elegans* can offer an intermediate view between cell culture and mammals. In these worms, it is possible to express WT or mutant human genes of interest in muscles and neurons (Caldwell et al., 2020; Eck et al., 2023). Moreover, it has been described that the replacement of a *C. elegans* homolog gene can be done with a single-copy knock-in of the WT or mutant human gene (Baskoylu et al., 2018). The expression of these human disease-associated genes can be inducible using inducible promoters or even by using protocols to trigger the aggregation of neurotoxic proteins in aging (Lim et al., 2020; Link et al., 2003; Wu et al., 2006). The study of synergies between distinct pathological proteinopathies may inspire the future of neurodegenerative disease models in *C. elegans* listed in Table 1.

**Table 1:** Neurodegenerative *C. elegans* models.

Disease	Protein	Transgene	Tissue expression	Phenotype	References
Alzheimer’s disease	APP	*apl-1*::SC_APP	Neuronal	Neurodegeneration	Yi et al. (2017)
	Aβ_1-42_	*unc-54*::Aβ_1-42_	Muscular	Movement deficit, Aβ_1-42_ aggregation	Link (1995)
		*unc-54*::Aβ_1-42_	Muscular	Movement deficit, Aβ_1-42_ aggregation	Fay et al. (1998)
		*unc-54*::Aβ_1-42_	Muscular	Movement deficit, Aβ_1-42_ aggregation	Fonte et al. (2002)
		*unc-54*::Aβ_1-42_	Muscular	Movement deficit, Aβ_1-42_ aggregation	McColl et al. (2012)
		*unc-54*::Aβ_1-42_	Muscular	Movement deficit, Aβ_1-42_ aggregation	Link et al. (2003)
		*snb-1*::Aβ_1-42_	Neuronal	Deficit on sensorimotor functions	Wu et al. (2006)
		*snb-1*::Aβ_1-42_	Neuronal	Deficit on sensorimotor functions	Link (2006)
		*unc-119*::Aβ_1-42_	Neuronal	Neurodegeneration	Fong et al. (2016)
		*eat-4*::Aβ_1-42_	Neuronal	Deficit on sensorimotor functions	Treusch et al. (2011)
	Aβ_1-42_ + tau	*aex-3*::tau; *snb-1*::Aβ_1-42_	Neuronal	Movement deficit	Benbow et al. (2020)
		*rab-3*::F3DK280; *snb-1*::Aβ_1-42_	Neuronal	Altered chemotaxis behavior, increases of protein aggregates	Wang C. et al. (2018)
Tauopathies	Tau	*rab-3*::F3DK280	Neuronal	Movement deficit, tau aggregation	Fatouros et al. (2012)
		*rab-3*::F3DK280(I277P)(I308P)	Neuronal	No obvious defect	Fatouros et al. (2012)
		*F25B3.3*::tau352PHP	Neuronal	Uncoordinated locomotion, incomplete neurite outgrowth	Brandt et al. (2009)
		*F25B3.3*::tau352WT	Neuronal	Uncoordinated locomotion	Brandt et al. (2009)
Parkinson’s disease	α-syn	*unc-51*::α-syn	Neuronal	Movement deficit, growth defect, impaired touch response	Kuwahara et al. (2008)
		*Punc-51*::α-syn (A30P)	Neuronal	Movement deficit, growth defect, impaired touch response	Kuwahara et al. (2008)
		*unc-51*::α-syn (A53T)	Neuronal	Movement deficit, growth defect, impaired touch response	Kuwahara et al. (2008)
		*unc-54*::α-syn:GFP	Muscular	Movement deficit, α-syn aggregation	Hamamichi et al. (2008)
		*unc-54*::α-syn:YFP	Muscular	Movement deficit, α-syn aggregation	van Ham et al. (2008)
	GFP	*dat-1*:GFP	Neuronal	Normal	Zhou et al. (2013)
Amiotrophic lateral sclerosis	SOD-1	*unc-25*::SOD1(G93A)::GFP	Neuronal	Motor neuron degeneration	Li and Le (2013)
		*snb-1*::SOD1(WT)::YFP	Neuronal	Motor deficits	Wang et al. (2009)
		*snb-1*::SOD1(G85R)::YFP	Neuronal	Motor deficits, synaptic transmission defects	Wang et al. (2009)
		*sod-1*::SOD-1(A4V)	Neuronal	Stress-induced cholinergic motor neuron degeneration	Baskoylu et al. (2018)
		*sod-1*::SOD-1(H71Y)	Neuronal	Stress-induced cholinergic motor neuron degeneration	Baskoylu et al. (2018)
		*sod-1*::SOD-1(L84V)	Neuronal	Stress-induced cholinergic motor neuron degeneration	Baskoylu et al. (2018)
		*sod-1*::SOD-1(G85R)	Neuronal	Stress-induced cholinergic motor neuron degeneration	Baskoylu et al. (2018)
		*sod-1*::SOD-1(G93A)	Neuronal	Stress-induced cholinergic motor neuron degeneration	Baskoylu et al. (2018)
	TDP-43	*snb-1*::TDP-43(WT)	Neuronal	Motor deficits	Liachko et al. (2010)
		*snb-1*::TDP-43(A315T)	Neuronal	Motor deficits, motor neuron degeneration	Liachko et al. (2010)
		*snb-1*::TDP-43(M337V)	Neuronal	Motor deficits, motor neuron degeneration	Liachko et al. (2010)
		*unc-47*::TDP-43(A315T)	Neuronal	Motor neuron degeneration	Vaccaro et al. (2012)
Huntington’s disease	HTT	*Osm-10:: Htt-polyQ*	Neuronal	Neurodegeneration	Faber et al. (1999)
		*mec-3*:: Htt-polyQ::GFP	Neuronal	Neurodegeneration	Parker et al. (2001)
		*unc-54*::GFP-Htt-polyQ	Muscular	Movement deficit	Nedosekin et al. (2021)
		*unc-54*::GFP-Htt-polyQ	Muscular	Movement deficit	Satyal et al. (2000)

Briefly, to carry out the models and express the various genes associated with each disease, the generation of *C. elegans* transgenic strains consists of the addition of a plasmid with a tissue-specific promoter and the gene with the sequence of interest. This plasmid is delivered by gonadal microinjection along with a marker gene to aim for the selection of transgenic animals. For example, the pRF4, which expresses a mutant collagen gene that induces a “roller” phenotype (Link, 1995), or the mtl-2/GFP plasmid, which results in constitutive intestinal expression of GFP (Fay et al., 1998), among others.

### 2.1 Alzheimer’s disease models

AD is the most common neurodegenerative disease in the aged population, characterized by progressive and irreversible memory loss (Breijyeh and Karaman, 2020). Although the molecular mechanisms underlying this pathology have not been fully elucidated, the known histopathological hallmarks of AD involve the presence of aggregated Amyloid-*β* (Aβ) peptide forming the extracellular amyloid plaques and the intracellular aggregates of hyperphosphorylated cytoskeleton-associated protein tau forming the neurofibrillary tangles in the brain (Selkoe and Hardy, 2016). Aβ accumulation is an outcome of sequential enzymatic processing of the human amyloid precursor protein (APP) by the membrane-bound enzymes β-secretase and the *γ*-secretase complex, which cleaved APP into several amino acid fragments such as Aβ_40_ and Aβ_42_ (Armstrong, 2009; Breijyeh and Karaman, 2020). There are several types of A*β* like monomers and soluble oligomers, including large and insoluble amyloid fibrils, which can accumulate to form amyloid plaques. This process induces tau phosphorylation and aggregation into neurofibrillary tangles (He et al., 2018; Link et al., 2003; Roda et al., 2022).

In *C. elegans*, the genome lacks both a clear β-secretase ortholog and an ortholog of APP. The APL-1 protein is an ortholog of the human amyloid beta precursor-like proteins 1 and 2 (APLP1 and APLP2), sharing a 71% of protein sequence identity to the intracellular domain of APP but without an Aβ domain (Sester et al., 2000). For that reason, AD models in *C. elegans* are primarily made by driving the expression of the Aβ peptide minigene, tau protein, or both (Griffin et al., 2017; Link, 1995), showing that *C. elegans* serves as an excellent model for studying AD by expressing human genes associated with the disease. However, an AD *C. elegans* model has been developed through the expression of human APP under a pan-neuronal promoter in a worm that expresses the Green Fluorescent Protein (GFP) in ventral cord (VC) cholinergic neurons (Pereira et al., 2015; Yi et al., 2017). In this model, the expression of APP induces neuronal damage, characterized by shrinking and dimming of GFP fluorescence in somas of VC neurons (Alvarez et al., 2022; Yi et al., 2017). Given the lack of a β-secretase to process APP into Aβ in *C. elegans*, these results indicate that APP may lead to neurodegeneration in these worms without Aβ production; however, we cannot discard APP processing in worms by other enzymes generating toxic products. Thus, the mechanisms of neurodegeneration induced by APP require more careful studies.

The main AD models are driven by muscle cell expression of Aβ using the *unc-54* promoter, the promoter of myosin in the body wall muscles (Fay et al., 1998; Griffin et al., 2017). It has been described that Aβ expression induces progressive paralysis and the localization of aggregates in body-wall muscles, detected by immunohistochemistry or staining procedures (Dostal and Link, 2010; Fay et al., 1998; Fonte et al., 2002; Link, 1995; Styren et al., 2000). The main muscular expression strains have promoters with constitutive expression; including CL2006, CL2008, CL2120, and GMC101 strains (Fay et al., 1998; Link, 1995, 2006; McColl et al., 2012). Even though constitutive expression is useful, some physiological parameters like paralysis have shown an incomplete penetrance and different ages of onset (Alvarez et al., 2022). Due to that, strains have been developed with a temperature-induced expression of Aβ under myo3 promoter, such as CL4176 (Link, 2006; Link et al., 2003; McColl et al., 2012). The increase of temperature from 16°C to 23°C leads to a clear fast paralysis phenotype, which can be easily scored and quantified in 100% of individuals. This inducible system is useful to get an insight into different states before and after Aβ expression (Link, 2006). On the other hand, the GMC101 strain showed a very useful phenotype. It expresses Aβ constitutively but a shift from 20°C to 25°C for 48 h triggers the paralysis phenotype (McColl et al., 2012). The rapid paralysis induced by Aβ_1-42_ in this strain is well suited for assessing drug effects to identify new protective compounds. However, while muscular expression is useful, it does not represent the AD pathology which affects neuronal viability. The strains CL2355 and CL2241 (Link, 2006; Wu et al., 2006) employ the inducible promoter of the *C. elegans* synaptobrevin ortholog (*snb-1*) to drive pan-neuronal expression of Aβ_1-42_. These strains are characterized by a reduced chemotaxis index and the oligomerization of Aβ is induced after upshifting temperature to 36°C (Wu et al., 2006). On the other hand, in order to analyze a long-term progressive neuronal dysfunction, the strain GRU102 expresses constitutively Aβ_1-42_ under *unc-119* pan-neuronal promoter. The Aβ_1-42_ expression is detectable by Western blot 12 days post-hatching and induces a decrease in longevity and sensorimotor functions in an age-dependent manner, resembling AD (Fong et al., 2016). This middle-age-onset appearance of the behavioral phenotypes allows the analysis of AD in *C. elegans* in an aging-dependent context.

Although the causes of AD are not clearly defined, there is a predictive association with allelic variants of the gene encoding Apolipoprotein E (APOE). While the presence of the APOEε4 allele increases the risk, APOEε2 has been identified as a protective factor against AD (Corder et al., 1994; Li et al., 2020; Talbot et al., 1994). *C. elegans* with glutamatergic neuronal expression of Aβ, crossed with *C. elegans* expressing the allele APOEε2, showed an increase in 30% of animals with all the glutamatergic neurons conserved, indicating an attenuation of Aβ-mediated toxicity. However, APOEε4 expression did not impact neurodegeneration (Griffin et al., 2019), indicating that only a part of the mechanisms that govern Aβ-APOE interactions are conserved between worms and mammals. This could be useful for future studies to comparatively dissect how the APOE alleles interact with Aβ to drive neurodegeneration and neuroprotection.

Tau protein is also a key component of the neurodegenerative cascade in AD. *C. elegans* has a homolog of the tau protein as well as a homolog of other microtubule-associated proteins called PTL-1. However, its expression is restricted, and its mutations do not generate aggregation and neurodegeneration (Goedert et al., 1996; Gordon et al., 2008). Thus, the *C. elegans* models for studying tau pathology are made using human tau. The strains that have pan-neuronal expression of WT tau and tau carrying the mutations P301L and V337M have shown a progressive uncoordinated motor phenotype, while at the cellular level, axons display tau aggregates followed by axon degeneration (Kraemer et al., 2003). Moreover, tau P301L and V337M nematodes showed hyperphosphorylation of tau at many sites such as Serine 422 and the known epitope AT-8 (Kraemer et al., 2003). The increase of tau phosphorylation in specific positions is considered one of the earliest signs of neuronal degeneration and appears to precede tau aggregation (Braak et al., 1994). To analyze the role of AD-related tau phosphorylation, it had been developed *C. elegans* strains which overexpress pseudophosphorylated tau in specific sites in the nervous system (strain VH254), and its control, which overexpress fetal human tau (Strain VH255) (Brandt et al., 2009). Even though both strains show progressive age-dependent uncoordinated locomotion, only VH254 evidence gaps in the dorsal cord, indicating an incomplete neurite outgrowth or axonal pathfinding (Brandt et al., 2009). Furthermore, a strain has been developed with a deletion of K280 in tau protein, which induces tau aggregation (BR5270 strain), and a strain with the I277P mutation that prevents beta-sheet structure formation (BR5271 strain), reducing the aggregation. The pro-aggregation strain showed accelerated tau aggregation along with severely impaired motility and neuronal dysfunction, in opposition to the anti-aggregation strain (Fatouros et al., 2012).

The co-expression of Aβ and tau is also available in *C. elegans*, giving a better comprehensive view of pathological mechanisms using only one model. The *C. elegans* strain CK1609 is a crossbreed between CK1441 and CL2355, expressing human tau pan-neuronally under the control of *aex-3* promoter (Iwasaki et al., 1997), and Aβ_1-42_ in a temperature inducible manner (Wu et al., 2006). This model shows reduced locomotion compared to controls in liquid as well as solid media, and a compromised nervous system (Benbow et al., 2020; Kraemer et al., 2003; Sarparast et al., 2023; Wu et al., 2006). Furthermore, the number of mechanosensory neurons decreases, indicating severe neuronal dysfunction in this double-expression model. The analysis of the common sites of hyperphosphorylation widely demonstrated in AD and other tauopathies shows that p-Ser396/Ser404, p-Ser202, and p-Thr231 are not modified by Aβ expression. However, the expression of tau increased total Aβ levels in comparison with the strain that only expresses Aβ (Benbow et al., 2020). Moreover, another variant of this mixed model is the UM0001 strain which is the crossbreed between the CL2355 strain with the pro-aggregation tau strain BR5270 (Fatouros et al., 2012; Wang C. et al., 2018). This strain showed a decreased lifespan and altered chemotaxis behavior, as well as an increase in the number of total aggregates compared to control worms. To analyze the influence of tau on Aβ aggregation it was generated the UM0002 strain, which expresses Aβ and the anti-aggregation tau (BR5271), which shows a decrease in total aggregate formation (Fatouros et al., 2012; Wang C. et al., 2018), indicating that the aggregation of tau could have a role as a potentiator of Aβ expression and aggregation. These models of double expression refine the strategies for understanding the pathological mechanisms and therapeutic drug discovery.

*C. elegans* models of AD are useful models to study the conditions that promote the aggregation or disassembly of Aβ since the system is not perturbed by the presence of a competitor’s endogenous protein. Similar is the case for tau, where endogenous tau-like protein does not form aggregates that could lead to neurodegeneration. Moreover, the high homology between worms and mammals’ genes has led to the study of the different signaling pathways driving neurodegeneration induced by Aβ as well as the cellular responses to Aβ. For example, *C. elegans* models have been useful in describing the role of mitophagy (Fang et al., 2019), the mitochondrial unfolded protein response (UPR^mt^) (Sorrentino et al., 2017), insulin signaling (Cohen et al., 2006), the role of the heat-shock protein family (Fonte et al., 2002; Link et al., 2003), cytosolic unfolded protein response (Joshi et al., 2021), acetylcholine signaling (Ahmad and Ebert, 2017; Rebolledo et al., 2011), among several others. The simplicity of the model, along with the rapid growth and aging, has allowed the screening for several putative new therapeutic drugs (Kamal et al., 2024; Lublin and Link, 2013; Teo et al., 2020), as well as the screening of risk-associated genes (Apostolakou et al., 2021; Hudgins et al., 2024).

### 2.2 Parkinson’s disease models

PD is an age-related disease and the second most common neurodegenerative disorder affecting more than 10 million patients worldwide suggesting an increasing need for effective treatment for this disease (Balestrino and Schapira, 2020; Poewe et al., 2017). Patients develop motor symptoms such as rigidity, tremor, postural instability, and bradykinesia, resting tremors but also non-motor signs such as lack of motivation, depression, sleep disorders, loss of smell, and cognitive deterioration (Armstrong and Okun, 2020; Balestrino and Schapira, 2020; Bartels and Leenders, 2009; Halliday et al., 2011). These symptoms are derived from the death of dopaminergic neurons of the substantia nigra (SN) pars compacta, which regulate the integration and coordination of voluntary movement (Armstrong and Okun, 2020). Therefore, the death of dopaminergic neurons results in dopamine depletion along the entire nigrostriatal pathway, leading to the onset of motor symptoms (Fernandez and Chen, 2007). At the cellular level, there are found cytoplasmic inclusions called Lewis bodies (LBs) formed by the *α*-synuclein protein, being PD a synucleinopathy (Balestrino and Schapira, 2020). While the etiology is unknown in most patients, different genes have been identified to be related, such as *PARK1/SNCA,* encoding α-synuclein with the A53T mutation (Polymeropoulos et al., 1997), Parkin, ubiquitin carboxy-terminal hydrolase-1 (UCHL1), PTEN-induced kinase 1 (PINK1), Dai-suke-Junko-1, and leucine-rich repeat kinase 2 (LRRK2) (Gasser et al., 2011; Klein and Westenberger, 2012; Nalls et al., 2014).

The genome of *C. elegans* has orthologs of many genes associated with PD, with the important exception of PARK1/SNCA. Therefore, *C. elegans* does not express α-synuclein, being a suitable model for overexpression without endogenous interference (Caldwell et al., 2020). In addition, *C. elegans* is also ideal for such analysis due to the presence of well-described 8 dopaminergic neurons: 6 in the anterior and 2 in the posterior part of the body (Cooper and Van Raamsdonk, 2018). This facilitates rapid scoring of dopaminergic neurodegeneration during normal worm aging.

Given that the aggregation of α-synuclein is a key cellular and biochemical hallmark of PD, several *C. elegans* models have been developed expressing human SNCA linked to a fluorescent protein such as GFP or Yellow Fluorescent Protein (YFP) in order to easily analyze the aggregation of α-synuclein (Cooper and Van Raamsdonk, 2018). The expression of α-synuclein can be in body-wall muscles conducted by the *unc-54* promoter (NL5901 and OW13 strains) (Cooper and Van Raamsdonk, 2018; van Ham et al., 2008) which generates inclusions and aggregates in an age-dependent manner (Di Rosa et al., 2020; Hamamichi et al., 2008; van Ham et al., 2008). The expression in the muscle cells is particularly advantageous for the visual detection of *α*-synuclein due to the large of muscle cells and the strong expression driven by the *unc-54* promoter (Cooper and Van Raamsdonk, 2018).

On the other hand, there are also models with a pan-neuronal expression of α-synuclein under the control of the *unc-51* promoter or in dopaminergic neurons under the control of the *dat-1* promoter, which encodes to the sodium-dependent dopamine transporter (Kuwahara et al., 2008; Lakso et al., 2003; Robinson et al., 2017). Furthermore, *C. elegans* has also been used for the expression of mutated forms of α-synuclein carrying the A30P or A53P mutations driven by the *dat-1* promoter along with EGFP. The expression of these mutated forms induces the α-synuclein accumulation in somas, neuronal projections, and the loss of dopaminergic neurons (Kuwahara et al., 2006). Physiologically, these worms display an abnormal phenotype of food-sensing behavior, suggesting that mutated α-synuclein leads to neurodegeneration in dopaminergic neurons resembling PD pathology. Hence, this model allows the most robust loss of dopaminergic neurons and dopamine-dependent behaviors within a genetic context of PD (Hamamichi et al., 2008; Kuwahara et al., 2006).

In addition to genetic models, there are also several models based on toxins that affect specifically dopaminergic neurons. The most widely used PD model is the treatment with 1-methyl-4-phenyl-1,2,3,6-tetrahydropyridine (MPTP) and 6-hydroxydopamine (6-OHDA), as well as Rotenone and Paraquat (Gonzalez-Hunt et al., 2014; Meredith and Rademacher, 2011; Simola et al., 2007). They are selectively accumulated by dopamine neurons through presynaptic dopamine transporters (DATs), causing an increase in ROS and mitochondrial dysfunction (Simola et al., 2007). MPTP and 6-OHDA have been used in mice, cell cultures, and *C. elegans* (Braungart et al., 2004; Meredith and Rademacher, 2011; Sun et al., 2018). As in other species, the treatment of worms with MPP+ (1-Methyl-4-phenylpyridinium), the active metabolite of MPTP, and 6-OHDA lead to neurodegeneration of dopaminergic neurons in *C. elegans*, allowing the pharmacological modeling of PD pathology (Braungart et al., 2004; Morton et al., 2023; Nass et al., 2002). Moreover, the exposure of *C. elegans* to rotenone 4 μM induces the progressive loss of dopaminergic neurons (Zhou et al., 2013), as well as the exposure to mitochondrial genotoxic compounds such as manganese and Aflatoxin B1, which also leads to neurodegeneration in *C. elegans* (Chen P. et al., 2015). In all these studies, *C. elegans* strains, which express GFP under the control of the *dat-1* promoter, are the main tool established for several of these pharmacologic models of PD. In that way, the analysis can be performed through the quantification of the number of somas, the decline of GFP fluorescence on somas, or observing the progressive damage on dendrites from smooth dendrites to blebs formation and broken dendrites (Cooper and Van Raamsdonk, 2018; Fu et al., 2014; Zhou et al., 2013). All these results show a wide range of possibilities for studying PD in *C. elegans*, from genetics to pharmacological models.

PD models in *C. elegans*, similar to AD models, have allowed the understanding of the mechanisms of α-synuclein aggregation and the signaling pathways and genes involved in the specific neurodegeneration of dopaminergic neurons. Among others, these models have shown the involvement of endocytic pathways in α-synuclein neurotoxicity (Kuwahara et al., 2008; Nim et al., 2023; Wang et al., 2021), the role of LRRK2 in dopaminergic neurodegeneration (Liu et al., 2011; Naidoo and de Lencastre, 2024; Saha et al., 2009; Yao et al., 2010), and the role of different α-synuclein mutations and other protein regulators in its aggregation (Kuwahara et al., 2006; Perni et al., 2021; van Ham et al., 2008). Thus, *C. elegans* models are a powerful tool not only for the testing of different drugs and gene screening like in the case of AD, but also due to the facility to assess different mutations in PD-associated genes such as SNCA, LRRK2, and others.

### 2.3 Amyotrophic lateral sclerosis models

ALS is a neurodegenerative disease characterized by the selective death of upper motor neurons. The cell bodies of these motor neurons are present in the motor cortex and brainstem, or lower, in the ventral horn of the spinal cord (Bruijn et al., 2004; Goodall and Morrison, 2006; Mancuso and Navarro, 2015; Pasinelli and Brown, 2006). The loss of motor neurons leads to muscle weakness, progressive atrophy, paralysis, and death within a period of 1–5 years from the onset of the disease (Kiernan et al., 2011). Its etiology is still unknown, but up to date, there are more than 45 human genes implicated as genetic drivers of familial ALS (fALS) (Smukowski et al., 2022). Among the most common proteins implicated are Cu/Zn-Superoxide dismutase 1 (SOD1), transactive response DNA binding protein 43 (TARDBP or TDP-43), Fused in sarcoma Translocated in sarcoma (FUS/TLS), and the hexanucleotide repeat expansions in C9ORF72 (Smukowski et al., 2022). Interestingly, 20% of fALS are associated with point mutations in the gene for the antioxidant enzyme SOD1 (Rosen et al., 1993). More than 200 SOD1 mutations have been described, which are characterized by a wide range of phenotypic aggressiveness (Bernard et al., 2020; Valentine and Hart, 2003), being the most typical mutations the G93A, D90A, and A4V (Pansarasa et al., 2018).

In the *C. elegans* genome, there are five SOD genes, *sod-1* and *sod-5* encode the Cu/Zn-SOD, *sod-2* and *sod-3* encode the Mn-SOD, and *sod-4* encodes an extracellular SOD (EC-SOD) (Eck et al., 2023). As the mutation SOD1(G93A) is commonly found in 20% of fALS patients, it has been widely used to develop ALS models in worms. The neuronal expression of SOD1(G93A) under the *unc-25* promoter leads to the expression in all 19 GABAergic motor neurons. The main characteristics of these worms are motor impairment, reduced lifespan, and SOD1 aggregation, sharing several features with fALS (Li et al., 2013; Li et al., 2014; Zhang et al., 2023). Moreover, there are other human SOD1-driven ALS in *C. elegans*, using the mutant SOD1(G85R) tagged with YFP, which was expressed pan-neuronally under the *snb-1* promoter. This model also induces motor dysfunction and motor neuron degeneration (Li and Le, 2013; Wang et al., 2009).

The models described above are examples of overexpression of mutations in a background of normal SOD1 expression, which did not allow the characterization of the effect of the loss of function of SOD1. The alignment of the SOD1 protein sequence between humans and *C. elegans* showed a 71% protein similarity (Baskoylu et al., 2018), allowing editing of the endogenous worm *sod-1* gene and inserting the mutations such as A4V (HA2631 strain), H71Y (HA2632 strain), and G85R (HA2633 strain) in a null *sod-1* background. These strains also express GFP under *unc-17* promoter labeling cholinergic neurons. The fluorescence analysis showed cholinergic neuron degeneration after oxidative stress induction when compared to control *sod-1* null, indicating a toxic gain of function of mutant SOD1 in cholinergic neurons (Baskoylu et al., 2018). On the contrary, the H71Y and G85R mutations as well as the loss of WT *sod-1* lead to glutamatergic neuron degeneration after stressing stimuli, indicating that degeneration of glutamatergic neurons is due to a loss of function of *sod-1* (Baskoylu et al., 2018). This single-copy/knock-in of ALS may help to understand the neurotransmitter-type specificity of ALS and how the loss and the gain of toxic function of SOD1 can contribute to ALS pathology (Eck et al., 2023).

Regarding TDP-43 models, since the loss of function in *C. elegans* TDP-43 homolog *tdp-1* does not cause motor deficits or neurodegeneration, but produces double-strand RNA (dsRNA) foci and sensitivity to oxidative stress (Eck et al., 2023), the ALS models based on TDP-43 are generated using the human WT or mutated TDP-43. The overexpression of human TDP-43 under the *snb-1* promoter, as well as the fALS associated-mutations G290A, A315T, and M337V in the C-terminus of TDP-43 results in motor deficits, motor neuron degeneration, lifespan reduction, TDP-43 aggregation, and pathological phosphorylation (Koopman et al., 2023; Liachko et al., 2010). To study the effect of mutant TDP-43 in the structure of the dorsal and ventral nerve cords of *C. elegans*, transgenic worms were crossed with nematodes that expressed the reporter transgene GFP under the control of the *unc-25* promoter (Cinar et al., 2005). Transgenic worms that express mutant TDP-43 showed the loss of motor neurons’ somas and gaps in neurites (Liachko et al., 2010; Vaccaro et al., 2012), suggesting that TDP-43 induces degeneration of motor neurons. It is interesting to mention that the study showing that the loss of *tdp-1* leads to the production of dsRNA foci (Eck et al., 2023) was the starting point of a future study where it was shown that the knockdown of TDP-43 in mice also generates dsRNA accumulation specifically in degenerating motor neurons, along with an increase of astrogliosis, showing a possible role of dsRNA in the pathogenesis of ALS (Milstead et al., 2023). This suggests that some findings in *C. elegans* could be important in the transfer of knowledge to mammalian models.

Altogether, SOD1 and TDP43 models recapitulate the motor neuron loss observed in ALS patients; overall, this evidence endorses that *C. elegans* can be an accurate ALS model for studying mechanisms in sALS and fALS. For example, using SOD1 and FUS *C. elegans* models it was described the involvement of autophagia (Baskoylu et al., 2022; Li et al., 2013; Xu et al., 2022) and granule stress pathways (Kassouf et al., 2023) in the rescue of motor deficits and aggregate formation. More recently, the overexpression of CDC-48.1, a protein essential for the elimination of misfolded proteins from the endoplasmic reticulum (Mouysset et al., 2006), rescues the motor deficits in SOD1(G85R) *C. elegans* model (Tsioras et al., 2023) suggesting that protein quality control is also involved in the pathology. Additionally, the expression of mutated ALS-associated proteins has been found to activate an innate immune response, leading to an increase in the expression of antimicrobial NLP-29, and the activation of TIR-1/Sarm1 immune pathway (Beers and Appel, 2019; Heneka et al., 2014; Veriepe et al., 2015). Moreover this models has been used to described the involvement of the DLK-1/MAP3K12 axonal regeneration pathway (Tossing et al., 2022), where in an ALS background, the knockdown of fsn-1/FBXO45, rpm-1/MYCBP2, or parp-2/PARP2 decreases axonal degeneration.

Importantly, ALS models of *C. elegans* have also allowed the identification of some kinases involved in TDP-43 phosphorylation and accumulation (Eck et al., 2021; Liachko et al., 2014; Taylor et al., 2018), proposing them as possible therapeutic targets in handling ALS progression. Thus, these finding established in *C. elegans* has been important in the study of the mechanism of pathogenesis of ALS as well as looking for potential therapeutic opportunities.

### 2.4 Huntington’s disease models

HD is an autosomal-dominant neurodegenerative disorder characterized by the degeneration of neurons in the basal ganglia starting with the gradual atrophy of the striatum. This neurodegeneration is highly selective for striatal GABAergic medium-sized spiny neurons (MSNs) (Cowan and Raymond, 2006; Gil and Rego, 2008), which causes uncontrolled and involuntary movements and a decrease in cognitive and emotional capacities (Jiang et al., 2023). Initially, patients suffer falls and clumsiness of some movements, which increase until they lose control of voluntary movement (Jiang et al., 2023). The symptoms are caused by abnormal repetitions of the CAG trinucleotide in exon 1 of the huntingtin gene (*htt*). Despite this repetition is present in healthy individuals, the number of repetitions is increased in HD patients, with a cutoff number of 35 repetitions for healthy people. This repetition leads to the production of polyglutaminated HTT and the formation of protein aggregates (Manoharan et al., 2016).

*C. elegans* can be a fast model for analyzing several fragments of HTT along with several polyglutamine (PolyQ) repetitions’ numbers and their involvement in aggregates’ formation. One of the first *C. elegans* models expresses 171 amino acids of human HTT with 150 repetitions of polyQ (HttQ150) in sensory neurons under the *osm-10* promoter. This HttQ150 expression leads to neurodegeneration and aggregate formation (Htt171 strain) (Faber et al., 1999). Moreover, the expression of HTT in the nervous system fused to GFP allows the visualization of aggregates in neurons (Caldwell et al., 2020). The promoter *mec-3* drives the specific expression of the *htt* gene in mechanosensory neurons. It has been described that the expression of 57 amino acids of the HTT protein (Htt57) with long polyglutamine tails (Q88 and Q128) leads to aggregate formation in axons, in comparison with the expression of short normal (Q19) repeats (Parker et al., 2001). Furthermore, the phenotype was correlated with axonal degeneration instead of cell death, suggesting loss of synaptic connection instead of neurodegeneration (Parker et al., 2001).

The *C. elegans* strain AM141 expresses in muscles a 40-glutamine repeats (Q40) tract fused to a YFP (YFP reporter), driven by a muscle-specific promoter *unc-54* (Nedosekin et al., 2021). This transgenic nematode model forms fluorescent protein aggregates in four longitudinal stripes of body-wall muscle and becomes progressively paralyzed as it ages, culminating in death at approximately 14 days of age (Nedosekin et al., 2021). Similarly, in *C. elegans* which expresses Htt Q82-GFP under the *unc-54* promoter (Morley et al., 2002; Satyal et al., 2000), the polyglutamine expansions with 82 residues induce fluorescent intra-myofibrillar foci that increase in number and brightness impacting worms’ longevity (Satyal et al., 2000).

Although *C. elegans* has not been a massively used model for HD research, these models still share the facility to screen for drugs that promote Htt disaggregation or improve the phenotypic outcome (Voisine et al., 2007). On the other hand, *C. elegans* gives the possibility to screen more easily the phenotypic effects of different forms of Htt protein with different lengths in the polyglutamine tails.

Altogether, this evidence shows how *C. elegans* provides a platform that accelerates the attainment of a more mechanistic understanding of how specific protein expression modulates neuronal degeneration and establishes *C. elegans* as a valuable pre-clinical model of neurodegenerative diseases to accelerate future drug discovery.

## 3 Genetic strategies to study neurodegeneration in *C. elegans*

As said before, one important advantage of *C. elegans* is that they are easily genetically manipulated, which allows the generation of the wide library of transgenic strains mentioned above. Moreover, the genetic manipulation of these nematodes has been used not only to generate transgenic strains but also to study different cellular mechanisms involved in different disease contexts (Baskoylu et al., 2018; Sorrentino et al., 2017).

### 3.1 Interference RNA (RNAi)

Much research has been done using different genetic strategies to mutate several genes in *C. elegans*. One of the most used techniques is the use of RNAi to study the loss of function of genes and even it has been used to do genome-wide screenings (Kamath and Ahringer, 2003). The incorporation of a double-stranded RNA (dsRNA) that interferes with the function of an endogenous gene produces a potent and specific loss of function by a mechanism of antisense hybridization between the injected RNA and the endogenous mRNA (Fire et al., 1998; Grishok, 2005; Kamath and Ahringer, 2003). This dsRNA can induce transcriptional (TGS) or post-transcriptional gene silencing (PTGS) (Sijen et al., 2001) where TGS involves the methylation of the promoters to decrease RNA synthesis, while PTGS involves the degradation of specific sequences of RNA (Sijen et al., 2001). This tool is simple, systemic, and heritable, which allows the mutation to be present in various generations (Conte et al., 2015; Grishok, 2005).

The delivery of RNAi into *C. elegans* could be done in different manners depending on the specific goal. It was reported that the microinjection of RNAi into the head or tail of the worm induces gene interference in the whole organism, as well as soaking worms in a solution of RNAi, which also has the same effect (Conte et al., 2015). However, nowadays it is known that the ideal target tissue for RNAi delivery is the intestine. Feeding *C. elegans* with *E. coli* bacteria containing RNAi is sufficient for its distribution to other somatic cells and even germinal cells (Conte et al., 2015; Tabara et al., 1998). Indeed, this delivery form of RNAi is the gold standard due to its many advantages. It is cheaper than microinjection or soaking since it does not need an *in vitro* synthesis of the dsRNA. Moreover, it is a simple form of delivery that is less laborious than microinjection allowing easier large-scale experiments with a high number of worms or/and a high number of tested genes, which can lead to a high number of samples for biochemical analysis (Conte et al., 2015; Kamath and Ahringer, 2003).

Despite the prior advantages mentioned, RNAi gene modification is not free of limitations. First, it takes a lot of work to clone the DNA fragment and transform bacteria to obtain the RNAi strain of interest (Kamath and Ahringer, 2003). Moreover, the bacteria used to be transformed with RNAi is different from that used to feed the worms. While OP50 *E. coli* is used for feeding, The HT115 (DE3) *E. coli* strain is used for transformation (Papic et al., 2018). This strain is resistant to tetracycline and has been widely used to produce dsRNA (Papic et al., 2018). It contains an IPTG-inducible promoter for the T7 polymerase gene and also is deficient in RNAse III, which is essential for the RNAi phenotype (Papic et al., 2018; Romano et al., 1993). These bacteria are grown on special NGM plates that contain IPTG and the antibiotic (Romano et al., 1993). Thus, it is important to keep in mind these considerations when using RNAi.

Regarding its usefulness, RNAi genetic modulation has several uses. For example, it is useful to determine whether a particular protein is involved in different neurodegenerative-associated phenotypes. For example, it is described that mitochondrial dysfunction is a classical hallmark of Alzheimer’s disease (Torres et al., 2021b); thus, a study performed a wide screening of genes affected in this disease and then used RNAi for silencing a specific gene to evaluate its importance in the AD phenotype (Sorrentino et al., 2017). Specifically, they silenced ATFS-1, the main transcription factor associated with the mitochondrial stress response Mitochondrial Unfolded Protein Response (UPR^mt^) in the GMC101 AD nematodes. The RNAi for *atfs-1* showed a decrease in maximal and basal respiration and an increase in Aβ aggregation and paralysis (Sorrentino et al., 2017), suggesting that the silencing of this protein worsened the AD phenotype and proposing that the increase of this protein could be a beneficial target for decreasing AD phenotype. Another example is the silencing of DRP-1 and other mitochondrial genes associated with mitochondrial morphology for evaluating its beneficial effect on the motor impairment and mitochondrial fragmentation in the AM141 *C. elegans* model of Huntington’s Disease, which is associated with polyglutamine aggregation (Machiela et al., 2021). Despite the RNAi against *drp-1* showing a slight increase in the lifespan and a decrease in the aggregation of polyglutamine in the AM141 worms, it did not have any beneficial effects on mobility, mitochondrial morphology, or function. In the same line, they show 25 RNAi clones that reduce mitochondrial fragmentation, eight of which resulted in amelioration of motor impairments, without any effect on polyglutamine aggregation (Machiela et al., 2021). Thus, RNAi screening in this case allows the analysis of the correlation between phenotypes and cellular impairments.

### 3.2 CRISPR/Cas9

Another genetic tool used in *C. elegans* is the clustered regularly interspaced short palindromic repeats (CRISPR)-Cas9 system. Cas9 is an endonuclease that was found in Archaea and bacteria, that cleaves dsDNA not in a specific DNA sequence but is led by the sequence of two small RNA molecules (Jinek et al., 2012). These two RNA are fused to form a chimeric single guide RNA (sgRNA of 20 bp), which in its 5′ end has the homolog sequence of the DNA of interest to be cleavage (Jinek et al., 2012); thus, it could be programmed to cleave any desired sequence by changing its 5′ end. Moreover, the Cas9 endonuclease must recognize a protospacer-adjacent motif (PAM) on the DNA of interest. Indeed, the recognition of the PAM by Cas9 occurs previous to the scanning of the match sequence for the sgRNA (Dickinson and Goldstein, 2016). As well as RNAi, the CRISPR system can be heritable, by injecting vectors containing the Cas9 and sgRNA in the germline. An SV40 nuclear localization signal could be added to the Cas9 DNA to be localized to the nucleus under a promoter that drives expression in the germline, such as *eft-3,* while the sgRNA containing the target sequence is under a pol III promoter (U6) (Friedland et al., 2013).

Once the dsDNA is broken, it is repaired by the endogenous repairing systems, which are used for generating genetic editing. For example, using non-homologous end joining (NHEJ) induces insertions and deletions near the cleavage site, which if it is done on the coding region could induce a codon stop by frameshifting (Dickinson and Goldstein, 2016). This induces loss of function mutations. Another repairing system is homology-directed repair (HDR), which by homologous recombination (HR), uses a complementary single-stranded DNA to a specific sequence as a template, which could have nucleotide changes or a new complete gene that is copied to the genome (Riesenberg et al., 2023). HDR is less efficient and effective than NHEJ, nevertheless, studies are trying to improve the efficiency of this technique (Riesenberg et al., 2023; Riesenberg and Maricic, 2018; Robert et al., 2015).

Unlike RNAi strategies, which silenced genes, the CRISPR system generates specific mutations or knock-ins of endogenous genes which also allows the evaluation of the relationship between a precise gene function and a certain neurodegenerative phenotype (Dickinson and Goldstein, 2016). For example, a study showed the effect of a single-copy knock-in of *sod-1* in ALS. They added, independently, using the CRISPR system some amino acid changes present in ALS patients in the *sod-1* gene, which induces gain or loss of function of this enzyme (Baskoylu et al., 2018). They showed that both phenotypes contribute to neurodegeneration in ALS but with different targets. The gain of function of *sod-1* induces the formation of SOD-1 inclusions in response to oxidative stress, which results in cholinergic neurodegeneration. On the other hand, the loss of function of *sod-1* induces hypersensitivity of glutamatergic neurons to oxidative stress, which leads to its neurodegeneration (Baskoylu et al., 2018). In another context, CRISPR has been widely used in PD to modulate the expression of the different key genetic factors involved in its pathogenesis. For example, it was found several single-nucleotide polymorphisms (SNPs) in the tyrosine nonreceptor kinase-2 (TNK2) in PD patients (Farlow et al., 2016). Thus, a study evaluates the neuronal effects of these SNPs *in vivo*, using the nematode *C. elegans*, since they have a TNK2 homolog, which is the *sid-3* gene. Using the CRISPR system they generate two strains that encode specific SNPs in conserved regions mimicking the one found in PD patients (Nourse et al., 2023). They showed that these SNPs increase the vulnerability to 6-OHDA toxin, suggesting an altered dopamine uptake system (Nourse et al., 2023).

Moreover, this genome editing system along with others such as microinjection, also allows the insertion of coding sequences for fluorescent proteins, mainly GFP, under the control of endogenous promoters to evaluate localization, promoter activity, and expression levels of endogenous proteins in different contexts (Dickinson and Goldstein, 2016). The use of fluorescent proteins is facilitated by the transparent body of these nematodes, which also allows the visualization of individual cells and developmental patterns as well as the use of optogenetic tools (Corsi et al., 2015). This useful tool has allowed the generation of several reporter strains (Kaymak et al., 2016), which are used to evaluate whether different treatments activate or inhibit specific processes or protein expression, where the expression of a fluorescent protein, mainly GFP, is controlled by a promoter of interest (Kaymak et al., 2016; Ray et al., 2014; Yoneda et al., 2004). For example, taking the mitochondrial dysfunction described earlier and the importance of UPR^mt^ in AD, there are specific reporter strains for UPR^mt^ associated with the promoters of chaperones Hsp60 and Hsp6 (Yoneda et al., 2004). In that context, it was reported that the expression of polyglutamine of a specific length (PolyQ40) in neurons induces this stress response measured as an increase in the GFP fluorescence of the worms (Berendzen et al., 2016). In the same way, it was described that paraquat, which induces PD, induces mitochondrial dysfunction and increases ROS production measured with a reporter strain for oxidative stress, where GFP protein expression is controlled by the endogenous promoter of *sod-3* (Ray et al., 2014).

Besides the reporter strains, the expression of fluorescent proteins in *C. elegans* has been also used to mark specific proteins or even neurons (Lakso et al., 2003; Nagarajan et al., 2015). For example, it is possible to evaluate the neuronal loss in PD in a transgenic *C. elegans* strain that contains the GFP expression under the promoter of DAT-1, inducing its expression only in dopaminergic neurons (BZ555) (Berkowitz et al., 2008; Lakso et al., 2003). Thus, the neuronal loss and changes in neuronal morphology in different contexts could be measured by the changes in green fluorescence under a microscope. On the other hand, the expression of fusion proteins is useful for studying protein localization or protein aggregation. For example, it is possible to study the aggregation process of *α*-synuclein in the nematodes using a strain that contains a fusion protein between YFP and α-synuclein (OW13), which allows the evaluation of the aggregated states of this protein under the microscope (Li et al., 2024b; Sanguanphun et al., 2023; van Ham et al., 2008).

Thus, this genetic strategy has different possible uses depending on the experimental design, however, it must be taken into account that this approach is more complex and laborious than RNAi.

### 3.3 Crossbreeding

One important benefit of using *C. elegans* for studying neurodegenerative diseases is their short life cycle, as well as short reproductive cycle and high size of progeny (Strange, 2006; Zhang et al., 2020). Due to these characteristics, the production of new transgenic strains could be done by the crossbreeding between two “parent” strains to obtain a certain model of neurodegeneration with, for instance, loss or gain of function of specific proteins to evaluate their contribution to the neurodegenerative phenotype. Even though it is simpler and faster than mating other organisms, it still has its critical issues. For example, for crossing two strains, is needed a hermaphrodite carrying one genotype, and a male carrying the other genotype of interest, however, male appearance has a low frequency, nearly 0.2% (Corsi et al., 2015). Thus, the first critical step of crossbreeding *C. elegans* is the male obtaining. For that purpose, it has been described that the appearance of males increases after stress conditions (Chasnov, 2013; Morran et al., 2009). For example, it is reported that a higher frequency of male occurs during and after dauer exposure, and also after L4 hermaphrodites are exposed for a short time to high temperatures (30–37°C) (Fay, 2013; Hodgkin, 1983; Morran et al., 2009).

Another important issue about crossbreeding *C. elegans* is that both genotypes need to be followed during the process. For example, some strains contain specific mutations or deletions that can be measured by PCR (Harris et al., 1996; Wu et al., 2018) or others that contain a fluorescent marker that allows their tracing by microscopy (Teo et al., 2019; Wu et al., 2006). However, it is important to mention that not all strains have an easy-tracker marker; thus, this is something that has to be considered.

**Figure 1:**
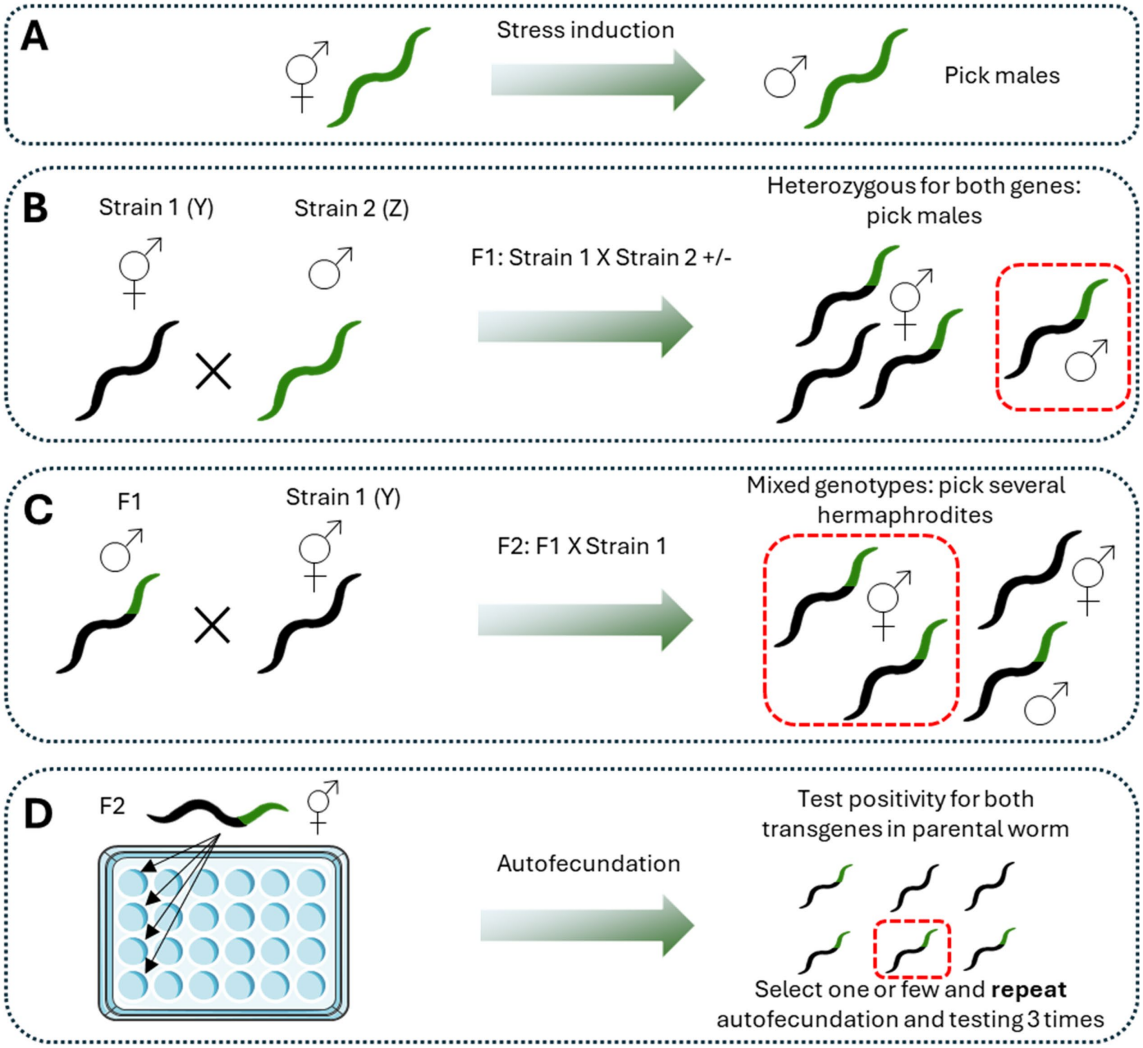
Procedure for crossbreeding. Crossbreeding between two different genotypes is made following a sequential process. The first step is the generation of male worms according to the desired method **(A)**. It is advantageous to choose the more easy and recognizable strain for that purpose. Then, favor the crossing by putting hermaphrodites of strain 1 (Y) with males of strain 2 **(B)**. All the F1 will be heterozygous for both transgenes. The third step is to pick males of the F1 and cross them with hermaphrodites of strain 1 (Y) **(C)**. Then, pick hermaphrodites positive for the most recognizable transgene and let them reproduce by self-fertilization. Discard the parental worm and let the progeny to growth. Test random worms for positivity in both transgenes **(D)**. Repeat this procedure until a homogenous population of worms positive for both transgenes is obtained. Figure designed and made with Servier Medical Art.

Once the previous considerations are taken into account, the process of outcrossing is quite simple (Figure 1). Males of one strain (Z) and hermaphrodites of the other strain (Y) are placed together in a 35 mm plate (with a 2:1 ratio) until the F1 is obtained (Slowinski et al., 2024). Since the fertilization of hermaphrodites by males results in a progeny of 50% male and 50% hermaphrodites (Morran et al., 2009), some males containing both markers are picked and mated again with hermaphrodites of the first strain (Y). After the F2 is obtained, several hermaphrodites containing the two markers are picked and placed on an individual plate for self-fertilizing. This procedure is repeated until 100% of the progeny contains both markers (Morran et al., 2009).

This procedure is useful, for example, for developing new transgenic strains with two phenotypes for modeling some pathology. For instance, the outcrossing of a strain expressing Aβ peptide with a strain expressing *α*-synuclein was performed to obtain a new transgenic strain with expression of both pathological proteins as a model of Lewis Bodies Dementia (LBD) (Huang et al., 2021). Moreover, the outcross between a strain expressing α-synuclein with one containing GFP on dopaminergic neurons allows the evaluation of neuronal degeneration associated with α-synuclein pathology, as well as the effect of some compounds on decreasing the α-synuclein-induced neurodegeneration (Nim et al., 2023). Furthermore, the outcrossing procedure can be used to evaluate the effect of the gain or loss of function of some protein in a reporter strain or a neurodegenerative disease model. For example, the outcrossing between a strain containing repeating glutamines (PolyQ) in the neurons and a reporter strain for the mitochondrial stress response UPR^mt^ can be used to evaluate the effect of aggregated proteins on this mitochondrial response (Berendzen et al., 2016). Also, a study showed the contribution of the specific protein SPR-4 to the Aβ-induced neurodegeneration by the outcrossing of an Aβ expressing strain with a mutant *spr-4* strain, showing that the loss of this protein increases significantly the Aβ-associated neurodegeneration (Lu et al., 2014).

Thus, there are different genetic strategies to manipulate the *C. elegans* genome in order to study the different pathways or specific proteins involved in the neurodegeneration associated with AD, PD, or HD, among others. Despite the use of one strategy over the other is dependent on the specific experiment and also the availability of the laboratory, all the described procedures are useful in the study of the underlying mechanism and the potential treatments of neurodegenerative disease.

## 4 *C. elegans* treatment with pharmacological agents

*C. elegans* are suitable tools in research not only for their easy genetic manipulation, as it was reviewed in the past section, but also because they are easily treatable with known pharmacological agents for the study of different signaling pathways.

### 4.1 Drug delivery

There are several standardized protocols according to the nature of the compounds used and the experimental design. A recent protocol paper was published as a detailed guide using compounds of different chemical nature (Wang et al., 2023). Among the different options, chemical compounds could be added to NGM agar before polymerization (1) being a constitutive part of the NGM; they could be added to NGM agar after polymerization (2) being absorbed by the solid NGM; they could be added to the liquid medium (3), or they could be mixed with live or dead OP50 bacteria (4) (Wang et al., 2023). Drug absorption using different delivery methods has been assessed using the water-soluble compound 5-fluoro-2′-deoxyuridine (FUDR) and the poor water-soluble compound Resveratrol. For both compounds with different solubilities in water, the rate of absorption by worms is similar. However, regarding the delivery method, treatment in liquid media or with killed bacteria reached the highest value of those compounds within worms (Zheng et al., 2013), suggesting that the delivery method is more important than the chemical nature when treating with pharmacological agents. However, another study showed that after testing 1,000 small-molecule compounds in high-throughput screening in worms, only 10% of the tested compounds were able to reach at least 50% of the concentration of the compound in the liquid media (Burns et al., 2010). These results showed that only a small percentage of the compounds with pharmacological potency reach important concentrations within the worms. Interestingly, the study that used FUDR and Resveratrol, on the other hand, suggests that their results showed comparable concentrations of compounds inside worms with concentrations obtained in mouse tissues (Zheng et al., 2013). Thus, while this is true for the tested compounds, the overall picture might indicate that these results could not be the norm, and the absorption of tested compounds should be tested as possible.

There are other less common and innovative forms of drug delivery in *C. elegans*. Liposome-mediated delivery of drugs has been successfully assessed with fluorescent dyes and compounds with different chemical properties (Martinez-Rodriguez et al., 2023; Roncato et al., 2019; Shibamura et al., 2009; Zhang et al., 2023). The method consists of the oral ingestion of the liposomes, mainly distributed in NGM or mixed with food. There are reports of a better delivery of fluorescent dyes compared to conventional methods of drug delivery (Shibamura et al., 2009). However, liposome-mediated delivery is not suitable for all types of compounds and proper delivery of the drug should be tested (Zhang et al., 2023). One interesting application is the delivery of the cyclic D, L-α-peptide CP-2, a peptide that modulates amyloid aggregates by interacting with soluble oligomers of Aβ, α-syn, and others (Richman et al., 2013). The mixing of liposomes containing CP-2 with dead bacteria improves the effectiveness of CP-2 alone in two Aβ-expressing worm strains, increasing longevity and chemotaxis and reducing Aβ oligomers (Senapati et al., 2024). Liposomes, however, have not been used exclusively for the delivery of chemical compounds or small peptides but also for whole proteins. Under this system, worms ingest the liposomes in liquid media. In the gut, liposomes protect the protein from the acidic environment, and the liposomes fuse with the gut lumen to release the protein, which diffuses throughout the body. Using this approach, an antibody against *α*-synuclein was successfully delivered, avoiding α-synuclein aggregation (Perni et al., 2017). Another non-conventional method is the use of nanoemulsions with the size of the particles ranging from 40 to 500 nm. This method was useful for delivering triglycerides by adding the nanoemulsion in the NGM (Colmenares et al., 2016). Similarly, the use of microparticles made of *γ*-cyclodextrin has been useful for delivering hydrophobic substances to *C. elegans* by oral ingestion. Importantly, the size of the particles should be similar to the size of bacteria, avoiding discrimination of worms during the intake (Kashima et al., 2012).

### 4.2 Methodological considerations

Regardless of the delivery method chosen, an important consideration is the food used in the studies that use pharmacological agents. For example, the election of the bacteria used could improve or impair the efficacy of the drugs tested (Ke and Aschner, 2019). The chemotherapeutic agent FUDR is widely used in research with *C. elegans* to avoid new progeny in a synchronized population (Mitchell et al., 1979). The use of *E. coli* OP50 improves the drug’s action, while the use of *Comamonas* impairs the drug’s action (Garcia-Gonzalez et al., 2017). In part, the effects of food in drug delivery are probably due to the action of the bacterial metabolism over the drug, producing more or less active metabolites or affecting bacteria-host interactions (Scott et al., 2017) since the reduction of *E. coli* infection to *C. elegans* increase the lifespan of the worms (Garigan et al., 2002). For example, Metformin, the most common drug used for type 2 diabetes mellitus (Rios et al., 2024), showed an effect in *C. elegans* depending on bacteria. The effect of Metformin in increasing worms’ longevity is absent when nematodes are grown in axenic cultures or cultures with dead bacteria (UV-irradiated) (Cabreiro et al., 2013). Moreover, metformin affects bacterial folate metabolism, decreasing its growth rate and affecting the methionine metabolism in the worms, acting as a mimetic of dietary restriction (Cabreiro et al., 2013). The effect described here is similar to the increase in worms’ lifespan due to dietary restriction, including the growth of worms in axenic cultures (Houthoofd et al., 2002). The effect of food sources on the effects of drugs in *C. elegans* is known and requires to be considered (Zhang et al., 2023). There are several ways to kill bacteria that are used as a food source. OP50 *E. coli* could be UV-irradiated, heat-killed, treated with antibiotics, or recently, it has been proposed the use of OP50 treated with paraformaldehyde (PFA) (Ke and Aschner, 2019). It has been shown that heat-killed OP50 is a low-quality food source for *C. elegans* because it generates arrest of development in larvae state L1/L2, suggesting nutritional deficiency, which could be partially reverted with vitamin B2 supplementation (Qi et al., 2017). Despite this finding, several studies have used heat-killed bacteria as a food source after hatching, or also since the L4 stage or only during adulthood, to avoid development defects (Dominguez-Meijide et al., 2021; Mor et al., 2020). However, the potential nutritional deficiency during the treatment of worms should be a matter of discussion as a potential interactor with the effect of the drug tested. To avoid these interactions, Scott Leiser’s group has proposed using fixed *E. coli* with PFA 0.5% for 1 h to generate metabolically inactive bacteria while preserving the structure and maintaining them edible for worms. Nevertheless, worms still prefer live OP50, and the metabolome of these worms is still different from worms treated with live OP50 (Beydoun et al., 2021). In this sense, the method of administration and the interactions with food should be a subject of important consideration for study designs and the discussion of the results in the field.

Another important consideration with the use of pharmacological agents in *C. elegans* is the solvent selection, or at least keeping in mind the effect of the solvent. Dimethyl Sulfoxide (DMSO) is one of the most used solvents in biology due to its amphipathic behavior, which is useful for dissolved organic and inorganic non-polar compounds in liquid media (Pegg, 2007; Santos et al., 2003). Despite its wide use and its low toxicity, below 10% (v/v), DMSO has not been out of controversy due to its impact on gene expression and other biological functions (Majdi et al., 2017; Verheijen et al., 2019). In *C. elegans* research, DMSO is not innocuous. For example, DMSO 1% reduces the pharyngeal pump and alters the morphology of worms (Calahorro et al., 2021). When DMSO is used above the solidified agar, it reduces longevity drastically when used at 3%, although under 2%, it does not affect longevity. DMSO above 0.5% delays development and affects both body size and thrashing rate (AlOkda and Van Raamsdonk, 2022), indicative of altered animal physiology. However, the most recognizable effect of DMSO is the increased longevity observed when DMSO is added to the agar before solidification or in liquid media (Frankowski et al., 2013; Wang et al., 2010). This effect is obvious at 0.5 and 2% of concentration, while 5% is deleterious. Moreover, DMSO affects gene expression, upregulating *daf16* downstream genes (Wang et al., 2010). Other common solvents are alcohols like methanol and ethanol. Both solvents activate a stress response and decrease feeding activity at concentrations above 2% in worms (Thompson and de Pomerai, 2005). However, another study showed that at least when measuring motility, both methanol, and ethanol are well tolerated at low concentrations (1–2%), and both preserved worms’ motility better than DMSO at a 4% concentration (Katiki et al., 2011), indicating low toxicity. While ethanol has been shown to be toxic in worms and a good model to study ethanol intoxication at high (Davies et al., 2003; Sterken et al., 2021) and medium concentrations (Wu et al., 2020) displaying effects on gene transcription and motility, low ethanol doses normally used as a solvent seem innocuous (Calahorro et al., 2021). Methanol, on the other hand, has not been assessed as a solvent in pharyngeal pump, motility, or gene expression, even though it is widely used, especially for organic extracts from natural compounds (Cheon et al., 2017; Liu et al., 2021; Navarro-Hortal et al., 2022; Roncato et al., 2019; Yen et al., 2023). In this regard, a characterization of methanol as a vehicle in studies with worms is necessary.

The treatment of *C. elegans* with different drugs has been widely used as a tool to decipher signaling pathways’ involvement in neurodegeneration and to test new putative therapeutic agents. The selection of the most suitable method of drug delivery with the corresponding precautions for the vehicle used and feeding method should be considered carefully in the experimental design.

## 5 Examples of methodological approaches

**Figure 2:**
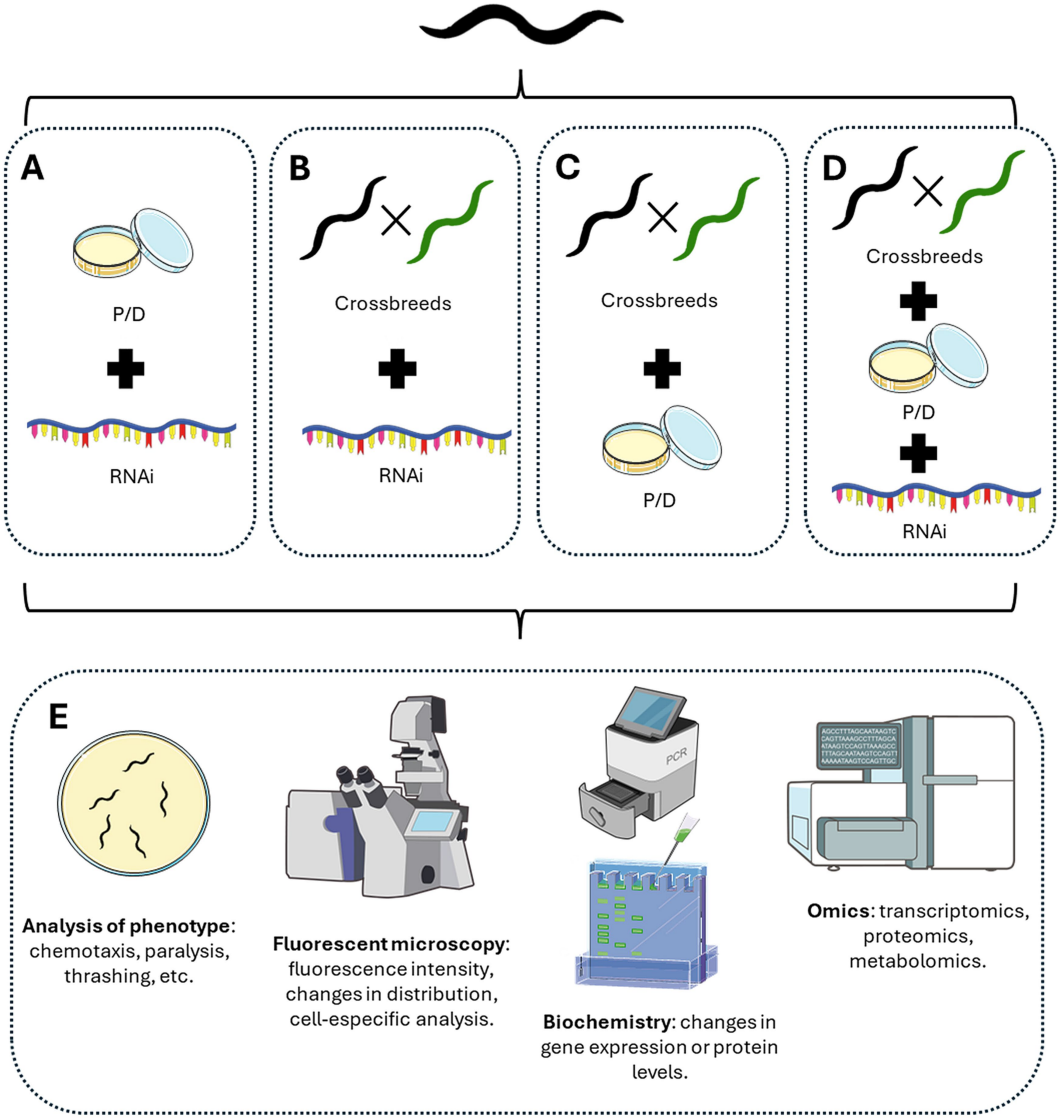
Different methodological approaches using *C. elegans* advantages. According to the goal of the experiments, different combinations of approaches could be performed. **(A)** Combining pharmacological treatment with RNAi-mediated knockdown. **(B)** Create different crossbreeds between *C. elegans* reporter strains and/or models of neurodegeneration with RNAi-mediated knockdown. **(C)** Create different crossbreeds between *C. elegans* reporter strains and/or models of neurodegeneration with pharmacological treatments. **(D)** Combined the generation of new crossbreeds with pharmacological and RNAi-mediated knockdown treatments. **(E)** Different experimental approaches could be tested at the phenotypic level by analyzing worms’ behavior, the distribution of fluorescent proteins by microscopy, protein or RNA levels, or even omics analysis of the genome, transcriptome, proteome, or metabolomic levels. Figure designed and made with Servier Medical Art and NIH BioART.

Pharmacological agents or natural extracts have been widely used in different *C. elegans* models of neurodegeneration to assess their benefits in improving the phenotype in different neurodegenerative diseases. There are thousands of examples in the literature (Aaron et al., 2016; Cordeiro et al., 2020; Chalorak et al., 2021; Chen X. et al., 2015; Dominguez-Meijide et al., 2021; Garcia-Moreno et al., 2019; Li et al., 2024a; Malar et al., 2021; Perni et al., 2021; Pohl et al., 2019; Prasanth et al., 2024; Wang et al., 2022; Wang X. et al., 2018; Xu et al., 2022). In the context of PD, the group of Hongyu Li explored the effects of nicotine and probable mechanisms of action in the worm. They combined the pharmacological induction of PD phenotype using 6-hydroxydopamine (6OHDA) in the BZ555 strain that expresses GFP in dopaminergic neurons, with the treatment with nicotine both during the induction of neurodegeneration and then for 72 h with the nicotine in the plates. Using this combination of pharmacological treatments, they showed that nicotine ameliorates dopaminergic neuron degeneration, improves bending, and increases dopamine content. They also used the strain OW13, which expresses α-syn coupled to YFP in worms’ muscles. In this model, they showed that nicotine treatment decreased the number of α-syn aggregates, recovered lipid deposits, and decreased ROS production. To gain insights into the mechanisms of action of nicotine, they also used other *C. elegans* reporter strains to assess DAF-16 and SOD-3 induction. Thus, with a combination of different *C. elegans* strains, models, and pharmacological treatments, Li’s group provides evidence of the beneficial role of nicotine in PD phenotype and potential molecular targets of nicotine (Ullah et al., 2024). In another example, the group of Zhen-Qiang Zhang used a combination of pharmacological approaches to assess the role of Hederagenin, a triterpenoid isolated from Matoa fruit and other plants, in AD phenotype and its potential role as peroxisome proliferator-activated receptor alpha (PPARα) agonist. The authors used the *C. elegans* strains CL4176 and GMC101, both muscle Aβ-expressing strains, and they delivered Hederagenin, as well as the PPARα agonist WY14643 and the PPARα antagonist MK-886 dissolved in M9 buffer using the NGM absorption method. They successfully showed the effect of Hederagenin in both paralysis and Aβ deposits, using the proper positive control for PPARα activation (WY14643), and the reverse in the effect of Hederagenin co-treating the compound with the corresponding antagonist (MK-886) (Xie et al., 2023). Thus, these authors successfully combined pharmacological strategies to link the phenotypic effect of the compound of interest with a known molecular target. However, these approaches are limited, and a better and deeper understanding of the molecular pathways involved in the effect of a certain drug or natural compound could be performed by combining both genetic and pharmacological techniques. In this sense, there are multiple options and experimental designs that could be adopted (Figure 2) to tackle the specific questions. The use of more complex experimental designs has become increasingly common in the past 15 years, accelerated by the simplicity of RNAi techniques, which have been adopted as a standard procedure in several laboratories that work routinely with worms (Baumanns et al., 2023; Haque et al., 2020; Joshi et al., 2021; Mor et al., 2020; Tossing et al., 2022; Zhang et al., 2018; Zhi et al., 2023). Interestingly, we are now witnesses to ambitious experimental designs combining all the advantages that *C. elegans* can offer, including transgenic models, newly generated strains by crossbreeding, RNA interference, and pharmacological treatments to look deeper into the molecular mechanisms of neurodegeneration and the mechanism of action of potential neuroprotective compounds (Guha et al., 2022; Machiela et al., 2021; Martinez et al., 2017; Veriepe et al., 2015).

The first example described here combines several different models and crossbreeds with RNAi and pharmacological approaches in a well-designed 2017 study. The authors used the AD strain GMC101, which expresses Aβ peptide in the muscle, and the control strain CL2122. With an RNAi vector against the transcription factor *atfs-1*, the author showed that inhibition of the UPR^mt^ stress response increased Aβ levels and worsened the AD-like phenotype of worms. Moreover, crossing GMC101 with worms that overexpress *atfs-1*, they generated the new strains AUW9 and AUW10, which showed reduced AD-like phenotype. Moreover, by using both RNAi and pharmacological treatments with Doxycycline, Nicotinamide Riboside, or Olaparib added to the NGM, they showed that UPR^mt^ induction in the GMC101 strain attenuated Aβ toxicity in the worms (Sorrentino et al., 2017). Thus, using a combination of techniques, the authors showed solid evidence for the role of UPR^mt^ in AD pathophysiology, a hypothesis that was subsequently tested in mice (Sorrentino et al., 2017). This study represents a good example of how well-designed studies in *C. elegans* led to valuable biological information replicable in other systems.

The final example here also used different techniques in a challenging research paper. The authors generated a transgenic worm expressing the mitophagy receptor DCT-1 fused to GFP and the autophagosomal protein LGG-1 fused with DsRed in neurons to assess mitophagy through co-localization (Fang et al., 2017). To assess mitophagy in an AD model, they crossbreed this mitophagy-sensor worm with the *C. elegans* strain CL2241, which expresses Aβ in all neurons. Using this crossbreed, they showed that mitophagy is impaired under normal conditions but also under stress conditions, treating worms with Paraquat. In this work, the authors used NGM supplemented with mitophagy inducers like Urolithin A and Nicotinamide Mononucleotide (NMN) to show the efficacy of these compounds as mitophagy inducers, but also they used this methodology to assess the efficacy of mitophagy inducers in ameliorating AD phenotype in the *C. elegans* strain CL2355 that also displays neuronal Aβ expression (Fang et al., 2019). This work used a sophisticated panel of experiments that combined different tools in *C. elegans* to support their hypothesis and conclusions on the role of mitophagy in AD pathology.

The combination of different strategies and tools like genetic manipulation, the generation of new strains, and pharmacological treatments demonstrate how advantageous is the nematode *C. elegans* as a biological model. Moreover, these are a powerful route to test new hypotheses, molecular mechanisms, and the study of putative new therapeutic strategies in biomedical research, especially in neurodegenerative diseases.

## 6 Conclusion

The use of *C. elegans* for the study of neurodegenerative diseases is suitable and quite advantageous in comparison to other animal models. Their easy maintenance, short life, short reproductive cycle, transparency, and easy genetic manipulation made these nematodes an outstanding biological model. There are a huge number of *C. elegans* models for AD, PD, ALS, and HD, which try to resemble human diseases and facilitate the study of their pathogenesis. However, despite the many advantages of *C. elegans*, it is important to be aware of the considerations of using this nematode, mainly when choosing the strains and the better strategy for each experimental design. Thus, in that way, it is possible to get the most out of the different tools that are available for the use of this nematode.
